# Bis[2-(1,3-dioxoisoindolin-2-yl)eth­yl] phthalate

**DOI:** 10.1107/S160053680905524X

**Published:** 2010-01-13

**Authors:** Kai Yang, Qiang Guo, Shi-Fan Wang

**Affiliations:** a School of Ocean, Hainan University, Haikou 570228, People’s Republic of China; b Experimental Teaching Center of Marine Biology, Hainan University, Haikou 570228, People’s Republic of China; c Key Laboratory of Tropical Biological Resources of Ministry of Education, Hainan University, Haikou 570228, People’s Republic of China

## Abstract

The title compound, C_28_H_20_N_2_O_8_, was synthesized by the reaction of isobenzofuran-1,3-dione and 2-amino­ethanol in a one-pot reaction. The benzene and five-membered rings are slightly twisted to each other, making dihedral angles of 2.77 (9) and 1.77 (10)°. The rings of the phthalimide groups make dihedral angle of 57.64 (7) and 83.46 (7)° with the central benzene ring. Weak C—H⋯O, C—H⋯π and π–π [centroid–centroid distance = 3.446 (1) and 3.599 (1) Å] inter­actions reinforce the cohesion of the crystal.

## Related literature

For a related structure, see: Liang & Li (2006 ▶). For bond-length data, see: Allen *et al.* (1987 ▶).
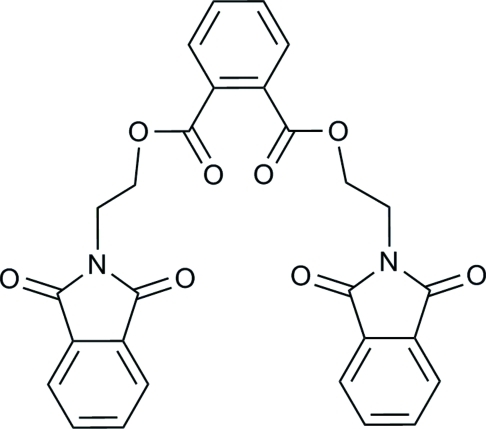


## Experimental

### 

#### Crystal data

C_28_H_20_N_2_O_8_
*M* *_r_* = 512.46
Monoclinic, 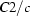

*a* = 15.021 (2) Å
*b* = 12.3953 (19) Å
*c* = 25.954 (4) Å
β = 90.125 (2)°
*V* = 4832.5 (13) Å^3^
*Z* = 8
Mo *K*α radiation
μ = 0.11 mm^−1^
*T* = 295 K
0.33 × 0.27 × 0.12 mm

#### Data collection

Bruker SMART CCD area-detector diffractometer
Absorption correction: multi-scan (*SADABS*; Bruker, 2002 ▶) *T*_min_ = 0.966, *T*_max_ = 0.988
18257 measured reflections
4718 independent reflections
3689 reflections with *I* > 2σ(*I*)
*R*_int_ = 0.031

#### Refinement

*R*[*F* ^2^ > 2σ(*F* ^2^)] = 0.043
*wR*(*F* ^2^) = 0.110
*S* = 1.03
4718 reflections
343 parameters
H-atom parameters constrained
Δρ_max_ = 0.15 e Å^−3^
Δρ_min_ = −0.18 e Å^−3^

### 

Data collection: *SMART* (Bruker, 2002 ▶); cell refinement: *SAINT* (Bruker, 2002 ▶); data reduction: *SAINT*; program(s) used to solve structure: *SHELXS97* (Sheldrick, 2008 ▶); program(s) used to refine structure: *SHELXL97* (Sheldrick, 2008 ▶); molecular graphics: *ORTEPIII* (Burnett & Johnson, 1996 ▶) and *ORTEP-32* (Farrugia, 1999 ▶); software used to prepare material for publication: *SHELXL97*.

## Supplementary Material

Crystal structure: contains datablocks global, I. DOI: 10.1107/S160053680905524X/dn2524sup1.cif

Structure factors: contains datablocks I. DOI: 10.1107/S160053680905524X/dn2524Isup2.hkl

Additional supplementary materials:  crystallographic information; 3D view; checkCIF report

## Figures and Tables

**Table 1:** Hydrogen-bond geometry (Å, °) *Cg*4 is the and *Cg*5 are the centroids of the C12–C17 and C22–C27 benzene rings, respectively.

*D*—H⋯*A*	*D*—H	H⋯*A*	*D*⋯*A*	*D*—H⋯*A*
C3—H3⋯O4^i^	0.93	2.39	3.315 (2)	174
C10—H10*B*⋯O5^ii^	0.97	2.55	3.163 (2)	121
C6—H6⋯*Cg*5	0.93	2.91	3.784 (2)	167
C19—H19*B*⋯*Cg*4^iii^	0.97	2.93	3.817 (2)	152

Symmetry codes: (i) 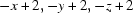; (ii) 
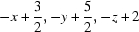
; (iii) 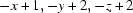.

**Fig. 1.**
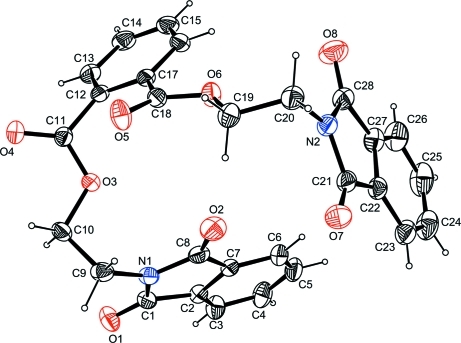
The asymmetric unit of (I) with the atom-labelling scheme. Displacement ellipsoids are drawn at the 30% probability level. H atoms are represented as small spheres of arbitrary radii.

## supplementary crystallographic information

### Comment

2-(2-hydroxyethyl)isoindoline-1,3-dione (Liang & Li, 2006) is a useful pharmaceutical intermediate in the synthesis of drugs containing aminoethyl group). The title compound, Bis(2-(1,3-dioxoisoindolin-2-yl)ethyl) phthalate includes two phthalamide groups and then easily provides two aminoethyl groups. As an intermediate for further synthesis, we obtained it in one-pot reaction.

The asymmetric unit is built up from a central phtalate with two pendant dioxoisoindolin groups (Fig. 1). The geometry of both the phthalimide rings compare well with the structure of the 2-(2-hydroxyethyl)isoindoline-1,3-dione (Liang & Li, 2006).

They could be regarded as planar with the largest deviation being 0.033 (2) Å and 0.028 (2) Å for C3 and C21 respectively, although the phenyl and the 5-membered rings are slightly twisted to each other making dihedral angles of 2.77 (9)° and 1.77 (10)° respectively. The phthalimide rings make dihedral angle of 57.64 (7)° and 83.46 (7)° with the central phenyl ring. Bond lengths and angles are normal and comparable to those observed in related compounds (Allen *et al.*, 1987).

Weak C-H···O, C-H···π and π-π interactions reinforce the cohesion of the crystal (Table 1,2).

### Experimental

44.5 g of isobenzofuran-1,3-dione (0.3 mol), 12.2 g of 2-aminoethanol (0.2 mol) were mixed in a flask and the mixture was heated up to boiling for one hour and then the reaction mixture was poured into water to give white precipitate. The precipitate was filtered, washed with water and dried in air to give the title compound as white solid (40.13 g, 78%). A little of the white solid was dissolved in mixed acetone/ water (50:1). After standing in air over a period of seven days, the acetone is evaporated, colourless crystals suitable for X-ray diffraction analysis were formed at the bottom of the vessel.

### Refinement

All H atoms were fixed geometrically and treated as riding with C—H = 0.93 Å (aromatic) or 0.97 Å (methylene) with U_iso_(H) = 1.2U_eq_(C).

### Crystal data

**Table:**

C_28_H_20_N_2_O_8_	*F*(000) = 2128
*M**_r_* = 512.46	*D*_x_ = 1.409 Mg m^−^^3^
Monoclinic, *C*2/*c*	Mo *K*α radiation, λ = 0.71073 Å
Hall symbol: -C 2yc	Cell parameters from 19894 reflections
*a* = 15.021 (2) Å	θ = 2.3–24.0°
*b* = 12.3953 (19) Å	µ = 0.11 mm^−^^1^
*c* = 25.954 (4) Å	*T* = 295 K
β = 90.125 (2)°	Block, colourless
*V* = 4832.5 (13) Å^3^	0.33 × 0.27 × 0.12 mm
*Z* = 8	

### Data collection

**Table:**

Bruker SMART CCD area-detector diffractometer	4718 independent reflections
Radiation source: fine-focus sealed tube	3689 reflections with *I* > 2σ(*I*)
graphite	*R*_int_ = 0.031
φ and ω scans	θ_max_ = 26.0°, θ_min_ = 1.6°
Absorption correction: multi-scan (*SADABS*; Bruker, 2002)	*h* = −18→18
*T*_min_ = 0.966, *T*_max_ = 0.988	*k* = −15→15
18257 measured reflections	*l* = −32→32

### Refinement

**Table:**

Refinement on *F*^2^	Primary atom site location: structure-invariant direct methods
Least-squares matrix: full	Secondary atom site location: difference Fourier map
*R*[*F*^2^ > 2σ(*F*^2^)] = 0.043	Hydrogen site location: inferred from neighbouring sites
*wR*(*F*^2^) = 0.110	H-atom parameters constrained
*S* = 1.03	*w* = 1/[σ^2^(*F*_o_^2^) + (0.0561*P*)^2^ + 1.0157*P*] where *P* = (*F*_o_^2^ + 2*F*_c_^2^)/3
4718 reflections	(Δ/σ)_max_ = 0.001
343 parameters	Δρ_max_ = 0.15 e Å^−^^3^
0 restraints	Δρ_min_ = −0.18 e Å^−^^3^

### Special details

**Table:**

Geometry. All e.s.d.'s (except the e.s.d. in the dihedral angle between two l.s. planes) are estimated using the full covariance matrix. The cell e.s.d.'s are taken into account individually in the estimation of e.s.d.'s in distances, angles and torsion angles; correlations between e.s.d.'s in cell parameters are only used when they are defined by crystal symmetry. An approximate (isotropic) treatment of cell e.s.d.'s is used for estimating e.s.d.'s involving l.s. planes.
Refinement. Refinement of *F*^2^ against ALL reflections. The weighted *R*-factor *wR* and goodness of fit *S* are based on *F*^2^, conventional *R*-factors *R* are based on *F*, with *F* set to zero for negative *F*^2^. The threshold expression of *F*^2^ > σ(*F*^2^) is used only for calculating *R*-factors(gt) *etc*. and is not relevant to the choice of reflections for refinement. *R*-factors based on *F*^2^ are statistically about twice as large as those based on *F*, and *R*- factors based on ALL data will be even larger.

### Fractional atomic coordinates and isotropic or equivalent isotropic displacement parameters (Å^2^)

**Table:**

	*x*	*y*	*z*	*U*_iso_*/*U*_eq_	
C1	0.97630 (11)	1.05121 (13)	0.91255 (6)	0.0416 (4)	
C2	0.97412 (11)	0.96188 (13)	0.87460 (6)	0.0418 (4)	
C3	1.03959 (12)	0.89040 (15)	0.85964 (7)	0.0553 (5)	
H3	1.0973	0.8957	0.8724	0.066*	
C4	1.01605 (15)	0.81098 (16)	0.82513 (7)	0.0650 (5)	
H4	1.0588	0.7616	0.8144	0.078*	
C5	0.93112 (15)	0.80299 (16)	0.80617 (7)	0.0640 (5)	
H5	0.9172	0.7483	0.7830	0.077*	
C6	0.86611 (13)	0.87458 (16)	0.82088 (6)	0.0548 (5)	
H6	0.8086	0.8695	0.8078	0.066*	
C7	0.88892 (10)	0.95428 (13)	0.85562 (6)	0.0400 (4)	
C8	0.83416 (10)	1.03777 (13)	0.88082 (6)	0.0431 (4)	
C9	0.86318 (12)	1.18167 (13)	0.94637 (6)	0.0481 (4)	
H9A	0.8060	1.2084	0.9345	0.058*	
H9B	0.9060	1.2399	0.9432	0.058*	
C10	0.85568 (11)	1.15007 (13)	1.00187 (6)	0.0459 (4)	
H10A	0.9140	1.1333	1.0159	0.055*	
H10B	0.8301	1.2085	1.0218	0.055*	
C11	0.75624 (10)	1.03503 (12)	1.04734 (6)	0.0388 (4)	
C12	0.70715 (10)	0.93102 (12)	1.04218 (6)	0.0389 (4)	
C13	0.73462 (12)	0.84711 (14)	1.07294 (6)	0.0501 (4)	
H13	0.7771	0.8590	1.0984	0.060*	
C14	0.69920 (14)	0.74499 (15)	1.06614 (7)	0.0579 (5)	
H14	0.7186	0.6882	1.0867	0.069*	
C15	0.63546 (13)	0.72739 (15)	1.02905 (7)	0.0553 (5)	
H15	0.6118	0.6587	1.0245	0.066*	
C16	0.60667 (11)	0.81130 (13)	0.99856 (7)	0.0475 (4)	
H16	0.5633	0.7991	0.9736	0.057*	
C17	0.64183 (10)	0.91381 (12)	1.00478 (6)	0.0382 (4)	
C18	0.60827 (10)	1.00635 (13)	0.97419 (6)	0.0402 (4)	
C19	0.53654 (12)	1.06028 (14)	0.89801 (6)	0.0475 (4)	
H19A	0.5853	1.1012	0.8833	0.057*	
H19B	0.4993	1.1091	0.9177	0.057*	
C20	0.48331 (11)	1.00681 (15)	0.85621 (6)	0.0492 (4)	
H20A	0.4368	0.9635	0.8718	0.059*	
H20B	0.4550	1.0618	0.8353	0.059*	
C21	0.59113 (10)	0.97752 (15)	0.78414 (6)	0.0430 (4)	
C22	0.63316 (11)	0.88163 (14)	0.76059 (6)	0.0462 (4)	
C23	0.68986 (12)	0.87307 (18)	0.71912 (7)	0.0602 (5)	
H23	0.7077	0.9336	0.7006	0.072*	
C24	0.71909 (14)	0.7715 (2)	0.70614 (8)	0.0773 (7)	
H24	0.7570	0.7632	0.6781	0.093*	
C25	0.69367 (17)	0.6827 (2)	0.73348 (9)	0.0850 (7)	
H25	0.7161	0.6154	0.7244	0.102*	
C26	0.63520 (16)	0.69062 (17)	0.77446 (8)	0.0742 (6)	
H26	0.6169	0.6299	0.7926	0.089*	
C27	0.60526 (12)	0.79191 (15)	0.78731 (7)	0.0518 (4)	
C28	0.54202 (12)	0.82721 (15)	0.82772 (6)	0.0509 (4)	
N1	0.89058 (8)	1.09290 (10)	0.91377 (5)	0.0400 (3)	
N2	0.53736 (9)	0.93881 (11)	0.82350 (5)	0.0434 (3)	
O1	1.03739 (8)	1.08221 (10)	0.93858 (5)	0.0612 (4)	
O2	0.75574 (8)	1.05580 (11)	0.87512 (5)	0.0634 (4)	
O3	0.79903 (7)	1.05658 (8)	1.00380 (4)	0.0446 (3)	
O4	0.76146 (8)	1.08763 (10)	1.08580 (4)	0.0541 (3)	
O5	0.61305 (9)	1.09848 (9)	0.98776 (5)	0.0592 (3)	
O6	0.57008 (8)	0.97564 (9)	0.93053 (4)	0.0485 (3)	
O7	0.59873 (9)	1.07155 (10)	0.77251 (5)	0.0588 (3)	
O8	0.50082 (10)	0.77394 (12)	0.85785 (5)	0.0771 (4)	

### Atomic displacement parameters (Å^2^)

**Table:**

	*U*^11^	*U*^22^	*U*^33^	*U*^12^	*U*^13^	*U*^23^
C1	0.0395 (9)	0.0390 (9)	0.0465 (9)	−0.0041 (7)	0.0018 (7)	0.0000 (7)
C2	0.0435 (9)	0.0440 (9)	0.0378 (8)	−0.0002 (7)	0.0015 (7)	0.0004 (7)
C3	0.0521 (10)	0.0608 (12)	0.0529 (10)	0.0127 (9)	−0.0041 (8)	−0.0104 (9)
C4	0.0805 (14)	0.0626 (13)	0.0519 (11)	0.0182 (11)	0.0013 (10)	−0.0143 (9)
C5	0.0878 (15)	0.0625 (13)	0.0419 (10)	−0.0062 (11)	0.0005 (10)	−0.0143 (9)
C6	0.0575 (11)	0.0690 (13)	0.0378 (9)	−0.0113 (9)	−0.0049 (8)	−0.0031 (8)
C7	0.0433 (9)	0.0447 (9)	0.0320 (8)	−0.0059 (7)	0.0008 (6)	0.0047 (7)
C8	0.0390 (9)	0.0513 (10)	0.0391 (8)	−0.0035 (7)	0.0028 (7)	0.0112 (7)
C9	0.0519 (10)	0.0333 (9)	0.0591 (10)	−0.0013 (7)	0.0144 (8)	−0.0001 (8)
C10	0.0465 (9)	0.0358 (9)	0.0554 (10)	−0.0100 (7)	0.0071 (7)	−0.0088 (8)
C11	0.0385 (8)	0.0411 (9)	0.0368 (8)	0.0018 (7)	0.0004 (6)	−0.0037 (7)
C12	0.0421 (8)	0.0395 (9)	0.0352 (8)	−0.0034 (7)	0.0096 (7)	−0.0016 (7)
C13	0.0574 (10)	0.0518 (11)	0.0413 (9)	−0.0048 (8)	0.0046 (8)	0.0062 (8)
C14	0.0731 (13)	0.0459 (11)	0.0546 (11)	−0.0041 (9)	0.0152 (10)	0.0147 (9)
C15	0.0646 (12)	0.0402 (10)	0.0612 (11)	−0.0135 (9)	0.0191 (9)	−0.0008 (8)
C16	0.0468 (9)	0.0438 (10)	0.0519 (10)	−0.0098 (8)	0.0090 (8)	−0.0076 (8)
C17	0.0383 (8)	0.0381 (9)	0.0383 (8)	−0.0036 (7)	0.0095 (6)	−0.0054 (7)
C18	0.0359 (8)	0.0421 (9)	0.0425 (8)	−0.0019 (7)	0.0047 (6)	−0.0087 (7)
C19	0.0502 (10)	0.0470 (10)	0.0452 (9)	0.0074 (8)	0.0007 (8)	−0.0030 (8)
C20	0.0397 (9)	0.0619 (11)	0.0460 (9)	0.0061 (8)	0.0016 (7)	−0.0046 (8)
C21	0.0392 (8)	0.0531 (11)	0.0366 (8)	−0.0003 (7)	−0.0047 (7)	0.0014 (8)
C22	0.0400 (9)	0.0600 (11)	0.0384 (8)	0.0027 (8)	−0.0047 (7)	−0.0034 (8)
C23	0.0461 (10)	0.0896 (15)	0.0448 (10)	0.0032 (10)	0.0008 (8)	−0.0081 (10)
C24	0.0588 (13)	0.116 (2)	0.0570 (12)	0.0185 (13)	0.0028 (10)	−0.0264 (13)
C25	0.0870 (17)	0.0921 (19)	0.0758 (15)	0.0372 (14)	−0.0090 (13)	−0.0328 (14)
C26	0.0930 (16)	0.0588 (13)	0.0709 (14)	0.0133 (12)	−0.0080 (12)	−0.0075 (11)
C27	0.0551 (10)	0.0538 (11)	0.0465 (9)	0.0073 (9)	−0.0039 (8)	−0.0060 (8)
C28	0.0574 (11)	0.0528 (11)	0.0424 (9)	−0.0031 (9)	0.0002 (8)	0.0036 (8)
N1	0.0381 (7)	0.0356 (7)	0.0463 (7)	−0.0017 (6)	0.0074 (6)	0.0001 (6)
N2	0.0437 (7)	0.0488 (8)	0.0378 (7)	0.0009 (6)	0.0018 (6)	−0.0028 (6)
O1	0.0471 (7)	0.0623 (8)	0.0743 (9)	−0.0038 (6)	−0.0092 (6)	−0.0211 (7)
O2	0.0369 (7)	0.0902 (10)	0.0630 (8)	0.0056 (6)	0.0004 (6)	0.0048 (7)
O3	0.0530 (7)	0.0394 (6)	0.0414 (6)	−0.0135 (5)	0.0080 (5)	−0.0058 (5)
O4	0.0636 (8)	0.0564 (8)	0.0423 (6)	−0.0081 (6)	0.0030 (6)	−0.0149 (6)
O5	0.0736 (9)	0.0393 (7)	0.0647 (8)	0.0074 (6)	−0.0174 (7)	−0.0136 (6)
O6	0.0586 (7)	0.0444 (7)	0.0424 (6)	−0.0010 (5)	−0.0060 (5)	−0.0055 (5)
O7	0.0657 (8)	0.0527 (8)	0.0579 (8)	−0.0033 (6)	0.0015 (6)	0.0094 (6)
O8	0.0953 (11)	0.0665 (9)	0.0696 (9)	−0.0120 (8)	0.0211 (8)	0.0121 (7)

### Geometric parameters (Å, °)

**Table:**

C1—O1	1.2014 (19)	C14—H14	0.9300
C1—N1	1.388 (2)	C15—C16	1.376 (2)
C1—C2	1.482 (2)	C15—H15	0.9300
C2—C7	1.374 (2)	C16—C17	1.385 (2)
C2—C3	1.380 (2)	C16—H16	0.9300
C3—C4	1.377 (3)	C17—C18	1.483 (2)
C3—H3	0.9300	C18—O5	1.1972 (18)
C4—C5	1.370 (3)	C18—O6	1.3250 (18)
C4—H4	0.9300	C19—O6	1.437 (2)
C5—C6	1.374 (3)	C19—C20	1.501 (2)
C5—H5	0.9300	C19—H19A	0.9700
C6—C7	1.380 (2)	C19—H19B	0.9700
C6—H6	0.9300	C20—N2	1.447 (2)
C7—C8	1.476 (2)	C20—H20A	0.9700
C8—O2	1.2077 (19)	C20—H20B	0.9700
C8—N1	1.383 (2)	C21—O7	1.209 (2)
C9—N1	1.448 (2)	C21—N2	1.389 (2)
C9—C10	1.497 (2)	C21—C22	1.479 (2)
C9—H9A	0.9700	C22—C27	1.376 (2)
C9—H9B	0.9700	C22—C23	1.378 (2)
C10—O3	1.4387 (18)	C23—C24	1.375 (3)
C10—H10A	0.9700	C23—H23	0.9300
C10—H10B	0.9700	C24—C25	1.365 (4)
C11—O4	1.1947 (18)	C24—H24	0.9300
C11—O3	1.3284 (18)	C25—C26	1.384 (3)
C11—C12	1.491 (2)	C25—H25	0.9300
C12—C13	1.374 (2)	C26—C27	1.375 (3)
C12—C17	1.395 (2)	C26—H26	0.9300
C13—C14	1.384 (3)	C27—C28	1.483 (2)
C13—H13	0.9300	C28—O8	1.197 (2)
C14—C15	1.374 (3)	C28—N2	1.389 (2)
			
O1—C1—N1	125.14 (15)	C15—C16—H16	119.8
O1—C1—C2	128.94 (16)	C17—C16—H16	119.8
N1—C1—C2	105.91 (13)	C16—C17—C12	119.25 (15)
C7—C2—C3	121.28 (15)	C16—C17—C18	121.21 (15)
C7—C2—C1	107.98 (14)	C12—C17—C18	119.48 (14)
C3—C2—C1	130.67 (16)	O5—C18—O6	123.45 (15)
C4—C3—C2	117.38 (18)	O5—C18—C17	124.08 (14)
C4—C3—H3	121.3	O6—C18—C17	112.45 (13)
C2—C3—H3	121.3	O6—C19—C20	106.72 (14)
C5—C4—C3	121.54 (18)	O6—C19—H19A	110.4
C5—C4—H4	119.2	C20—C19—H19A	110.4
C3—C4—H4	119.2	O6—C19—H19B	110.4
C4—C5—C6	121.02 (17)	C20—C19—H19B	110.4
C4—C5—H5	119.5	H19A—C19—H19B	108.6
C6—C5—H5	119.5	N2—C20—C19	112.51 (13)
C5—C6—C7	117.91 (17)	N2—C20—H20A	109.1
C5—C6—H6	121.0	C19—C20—H20A	109.1
C7—C6—H6	121.0	N2—C20—H20B	109.1
C2—C7—C6	120.87 (16)	C19—C20—H20B	109.1
C2—C7—C8	108.23 (13)	H20A—C20—H20B	107.8
C6—C7—C8	130.82 (15)	O7—C21—N2	124.87 (16)
O2—C8—N1	125.48 (16)	O7—C21—C22	129.12 (16)
O2—C8—C7	128.31 (16)	N2—C21—C22	106.00 (15)
N1—C8—C7	106.20 (13)	C27—C22—C23	121.37 (17)
N1—C9—C10	112.68 (13)	C27—C22—C21	108.09 (15)
N1—C9—H9A	109.1	C23—C22—C21	130.53 (18)
C10—C9—H9A	109.1	C24—C23—C22	117.4 (2)
N1—C9—H9B	109.1	C24—C23—H23	121.3
C10—C9—H9B	109.1	C22—C23—H23	121.3
H9A—C9—H9B	107.8	C25—C24—C23	121.4 (2)
O3—C10—C9	106.85 (13)	C25—C24—H24	119.3
O3—C10—H10A	110.4	C23—C24—H24	119.3
C9—C10—H10A	110.4	C24—C25—C26	121.4 (2)
O3—C10—H10B	110.4	C24—C25—H25	119.3
C9—C10—H10B	110.4	C26—C25—H25	119.3
H10A—C10—H10B	108.6	C27—C26—C25	117.4 (2)
O4—C11—O3	124.75 (15)	C27—C26—H26	121.3
O4—C11—C12	125.33 (15)	C25—C26—H26	121.3
O3—C11—C12	109.74 (12)	C26—C27—C22	121.03 (18)
C13—C12—C17	119.89 (15)	C26—C27—C28	130.66 (19)
C13—C12—C11	117.04 (15)	C22—C27—C28	108.31 (15)
C17—C12—C11	122.78 (14)	O8—C28—N2	125.08 (17)
C12—C13—C14	120.22 (17)	O8—C28—C27	129.23 (18)
C12—C13—H13	119.9	N2—C28—C27	105.69 (15)
C14—C13—H13	119.9	C8—N1—C1	111.67 (13)
C15—C14—C13	120.12 (17)	C8—N1—C9	124.20 (13)
C15—C14—H14	119.9	C1—N1—C9	124.13 (13)
C13—C14—H14	119.9	C21—N2—C28	111.88 (14)
C14—C15—C16	120.04 (16)	C21—N2—C20	123.93 (15)
C14—C15—H15	120.0	C28—N2—C20	124.20 (14)
C16—C15—H15	120.0	C11—O3—C10	118.61 (12)
C15—C16—C17	120.47 (17)	C18—O6—C19	116.31 (12)

### Hydrogen-bond geometry (Å, °)

**Table:**

Cg4 is the and Cg5 are the centroids of the C12–C17 and C22–C27 benzene rings, respectively.

**Table:**

*D*—H···*A*	*D*—H	H···*A*	*D*···*A*	*D*—H···*A*
C3—H3···O4^i^	0.93	2.39	3.315 (2)	174
C10—H10B···O5^ii^	0.97	2.55	3.163 (2)	121
C6—H6···Cg5	0.93	2.91	3.784 (2)	167
C19—H19B···*Cg*4^iii^	0.97	2.93	3.817 (2)	152

Symmetry codes: (i) −*x*+2, −*y*+2, −*z*+2; (ii) −*x*+3/2, −*y*+5/2, −*z*+2;  (iii) −*x*+1, −*y*+2, −*z*+2.

### Table 2 π–π interactions in (I)

*Cg*2 is the centroid of the N2/C21/C22/C27/C28 plane and *Cg*4 is the centroid of the C12–C17 phenyl ring

**Table:**

CgI—CgII	Cg–Cg (Å)	Cg—perp (Å)	Offset (°)	Symmetry
*Cg*2—*Cg*2	3.446 (1)	3.42	7.04	(1-x, y, 1/2-z)
*Cg*4—*Cg*4	3.599 (1)	3.355	21.2	(1/2-x, 1/2-y, -z)
